# Supplementary material for: GapPredict – A Language Model for Resolving Gaps in Draft Genome Assemblies
Source: IEEE/ACM Trans Comput Biol Bioinform. Author manuscript; Available in PMC 2022 Jan 20. (PMC8772386; doi:10.1109/TCBB.2021.3109557)
Supplement: supplemental material [file NIHMS1763064-supplement-supplemental_material.pdf]

Supplementary Material for: **Chen et al. (2021) GapPredict – A Language Model for Resolving Gaps in Draft Genome Assemblies**

Supplementary Figures

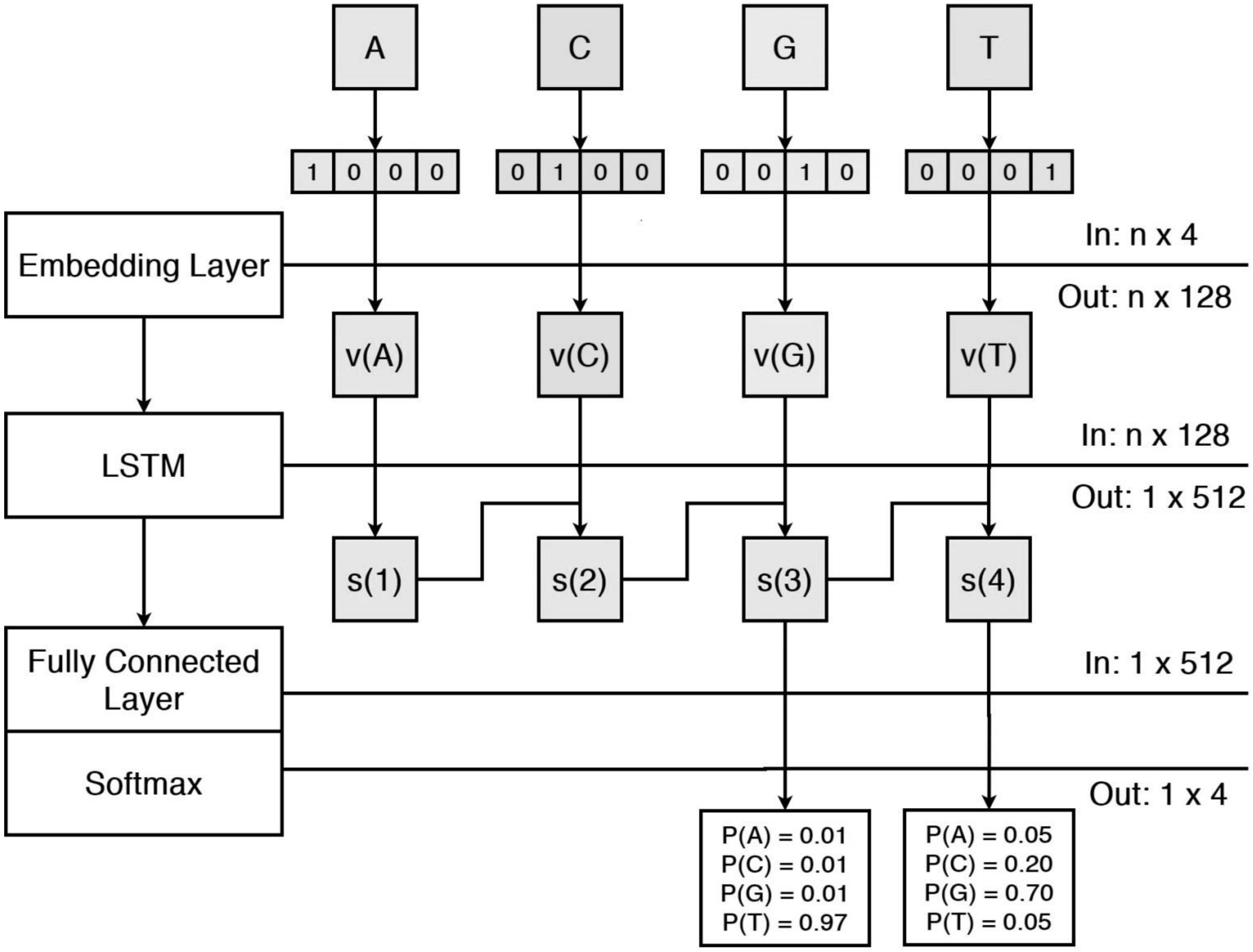

Fig. S1. Model architecture used by GapPredict. One-hot encoded bases first pass through an embedding layer, and the resulting vectors $v(b)$ (where b is the encoded base) are passed through an LSTM. The LSTM state $s(i)$ (where i is the iteration) can then be passed through a fully connected layer, normalized by the softmax function, to produce a probability vector P for the next base after a given input sequence. In this example, we pass in ACG as a seed. The GapPredict model predicts T as the most likely next base, so we add T to the input context to predict the base after.

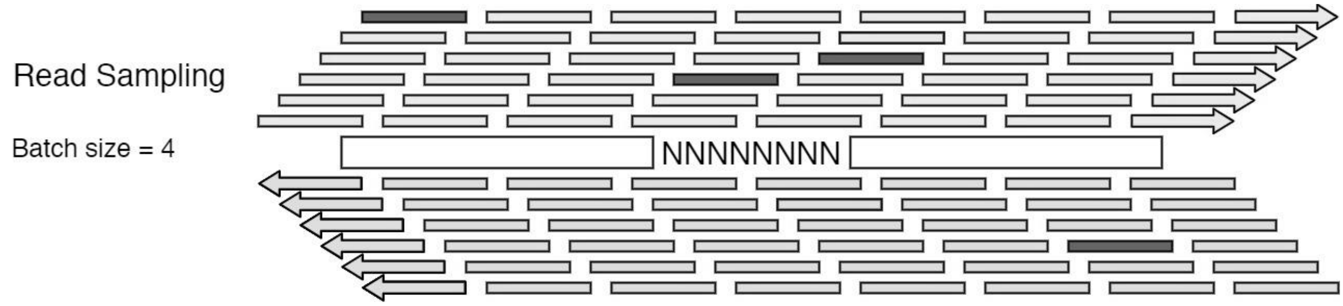

Fig. S2. Initial step of GapPredict's training protocol. In the first step, reads sampling, reads mapping to both the forward and reverse complement of the gap and its flanks are randomly sampled (dark grey) based on the batch size. This step and subsequent steps in GapPredict's training protocol are repeated for each iteration of a training epoch.

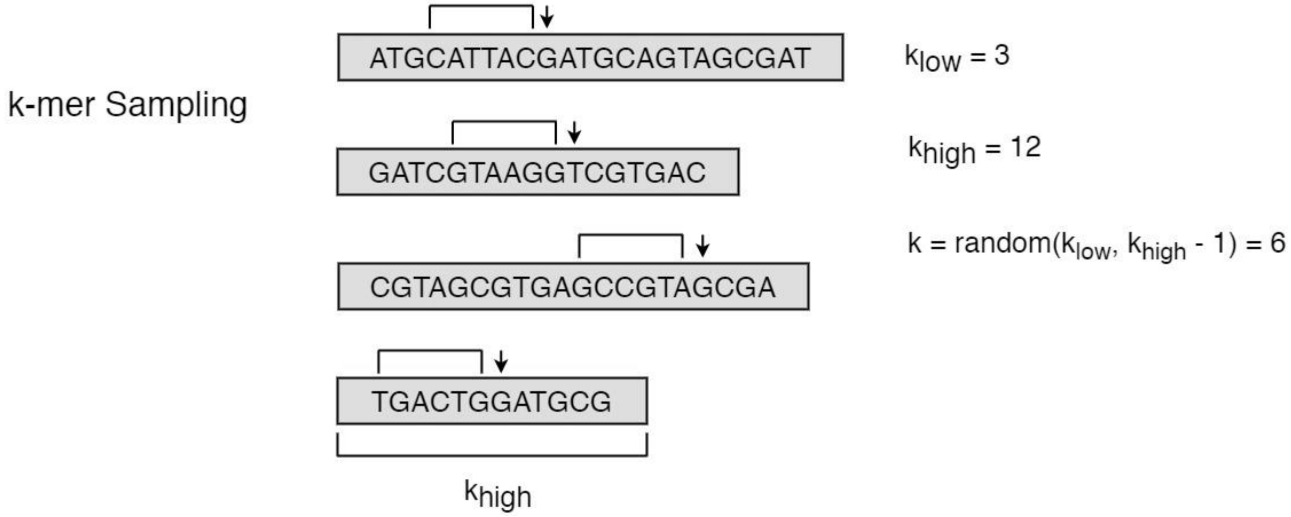

Fig. S3. Second step of GapPredict's training protocol. In the second step, k-mer sampling, from the reads sampled in the first step (Fig. S2), k_{high} is assigned to the minimum read length of the batch. We then randomly choose a length k between k_{low} (a hyperparameter) and $k_{high} - 1$ which is used to sample a subsequence from each read. The downwards arrow denotes the next base for each subsequence.

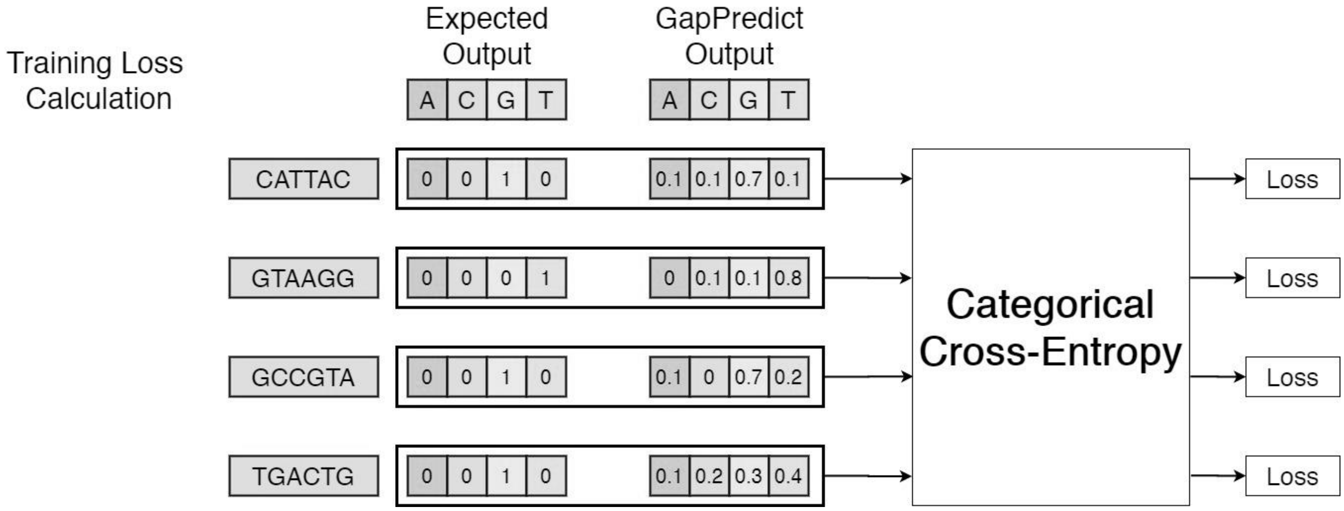

Fig. S4. Third step of GapPredict's training protocol. In the third step, training loss calculation, GapPredict predicts the next base given each subsequence sampled in the second step (Fig. S3). It then computes the categorical cross-entropy loss and adjusts its model's parameters according to the Adam optimizer.

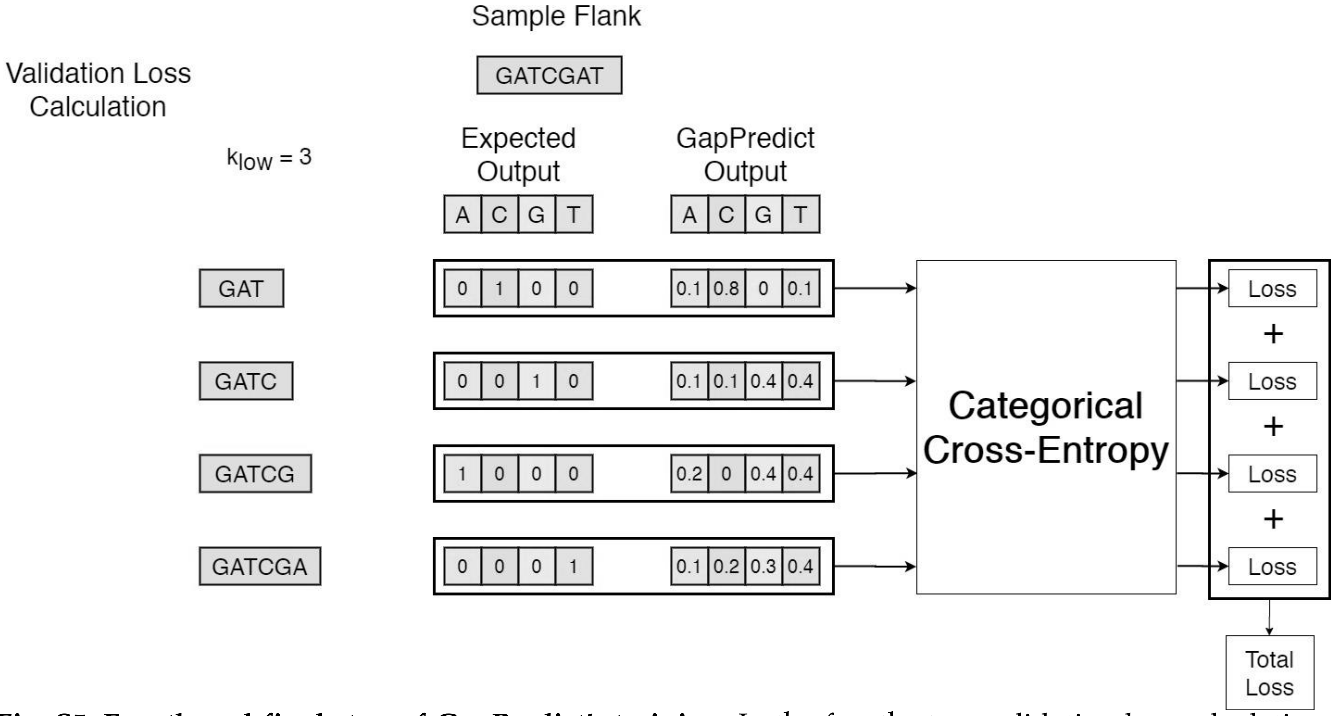

Fig. S5. Fourth and final step of GapPredict's training. In the fourth step, validation loss calculation, GapPredict obtains the left and right flanks for the gap and their reverse complement sequences. Then, for each of these four sequences, starting from a minimum index k_{low}, it iteratively predicts the next base for each subsequent index, given the preceding bases. The total validation loss for a flank is the sum of the validation loss for each predicted base. The total validation loss is the average of the validation losses for each flank. Following this step, we return back to the first step (Fig. S2) and sample a new batch until either the maximum number of epochs has been reached, or early stopping detects that our model has not improved over a number of epochs. In this example, our flank sequence is GATCGAT and $k_{low} = 3$, so we start with GAT and iteratively predict the next base until we reach the end of the flank.

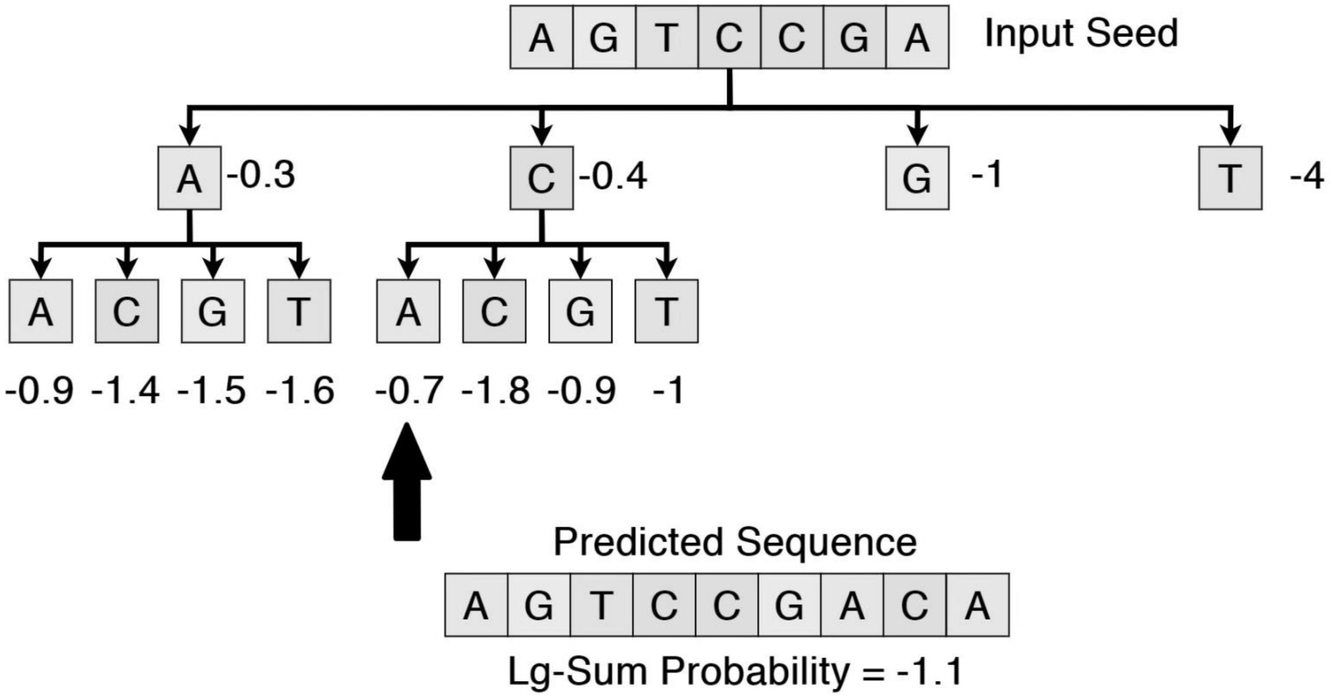

Fig. S6. Overview of the beam search algorithm used during the gap prediction stage. In this example, the beam width has been set to 2 and GapPredict will predict the next 2 bases. First, an input seed (eg. a flank, AGTCCGA in this example) is input into GapPredict and probability metrics (subjected to the logarithm function) for each of the four bases are output. In the first iteration, A and C are chosen as they have the least negative log-sum probability and the beam width allows at most 2 predictions for the next iteration. In the second iteration, probability metrics for each of the four bases are output given the input seed and the two predictions chosen from the previous iteration. AA and CA have the least negative log-sum probability and would be chosen for the next iteration. However, since we are only predicting the next 2 bases, we choose CA as it has the most optimal log-sum probability.

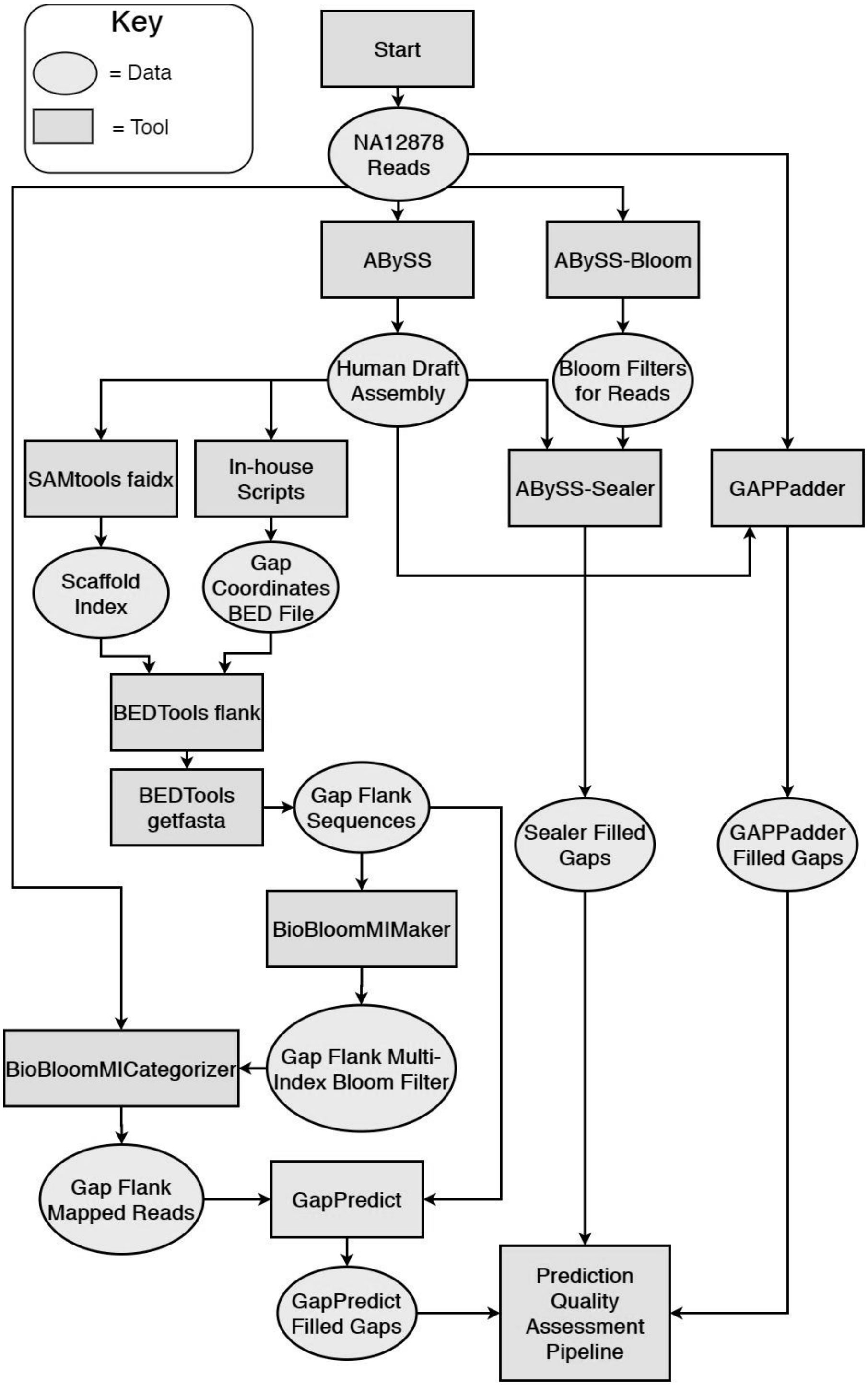

Fig. S7. Gap closing and analysis pipeline. The input to GapPredict (yellow box) includes sequences reads mapping to gap flanks for model training, and 500 bp of the left and right gap flanks for gap prediction after training. GapPredict outputs candidate sequences to fill gaps, which we compare with Sealer and GAPPadder's candidate sequences in the prediction assessment pipeline (red box).

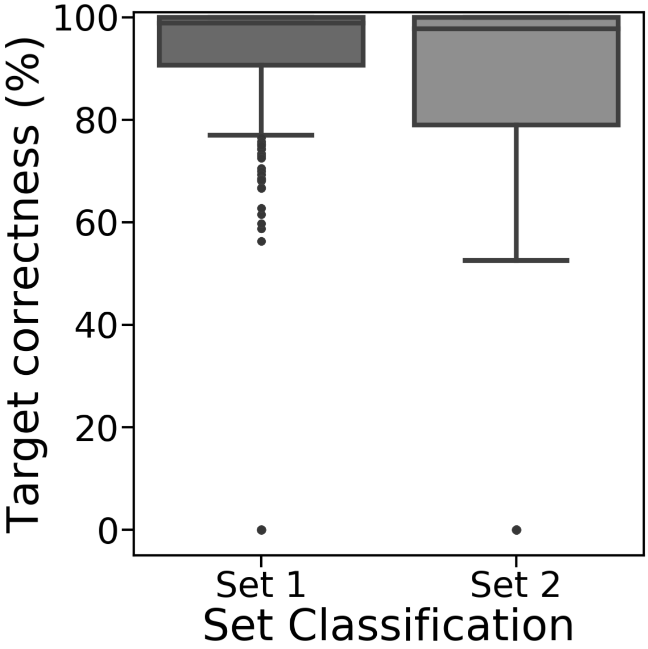

Fig. S8. Target percent correctness for gaps in set 1 (filled, n=868) and set 2 (unfilled, n=832) that were filled by GapPredict. Results from predictions using both flanking sequences as input are included, hence the sample sizes being double the number of gaps filled.

Supplementary Tables

Table S1. Summary metrics for target percent correctness among benchmarked tools. The mean and median target percent correctness for GAPPadder, Sealer, and GapPredict is shown in addition to the number of gaps underlying this data. Data for GapPredict predictions is also shown after grouping the predictions by type (i.e. "pass" or "fail", see Section 3.4 for definitions). Note: GapPredict produces two predictions per gap (one for each flanking sequence, on each DNA strand) and each corresponding prediction may "pass" or "fail" independently.

Tool	Set	# unique gaps filled	Total # of outputs	Target % correctness	
				Mean	Median
GAPPadder	1	425	425	83.8	99.0
	2	411	411	77.8	98.9
Sealer	1	430	430	88.7	98.9
	2	13	13	83.6	98.6
GapPredict	1 (all)	434	868	84.6	98.8
	1 (pass)	379	683	97.2	100.0
	1 (fail)	130	185	38.1	0.0
	2 (all)	416	832	76.8	97.7
	2 (pass)	326	543	96.9	100.0
	2 (fail)	199	289	39.0	0.0

Table S2. QUAST base accuracy metrics evaluating gap-filled sequence outputs of benchmarked tools against corresponding HG38 reference sequences. For this analysis, we selected gap sequences from set 1 (defined in section 3.1) that were successfully resolved by all three tools (367 out of 434 sequences satisfied this criterion). Gap sequence fraction refers to the percentage of aligned bases relative to the reference gap sequences. We highlighted the best results for each metric in bold face.

Tool	Gap sequence fraction (%)	# mismatches per 100 kbp	# indels per 100 kbp
GAPPadder	56.1	885.7	**80.1**
Sealer	**69.7**	**770.2**	87.3
GapPredict	56.9	1395.1	162.8

Table S3. Performance metrics for the benchmarked tools. The elapsed clock time and maximum memory usage of GAPPadder, Sealer, and GapPredict are shown. The metrics for Sealer and GAPPadder are for filling every gap in the draft assembly, whereas the metrics for GapPredict are for the 434 set 1 gaps and 416 set 2 gaps only. GapPredict's clock time is based on a single GPU; however, we lowered it by a factor of 4 by using 4 GPUs to each run a GapPredict instance.

Tool	Clock Time Elapsed (h)	Maximum Memory (GB)
GAPPadder (all gaps)	205	3
Sealer (all gaps)	2	52
GapPredict (set 1 and 2 gaps)	850	10 (GPU)
